# Fragmentation, integration and macroprudential surveillance of the US financial industry: Insights from network science

**DOI:** 10.1371/journal.pone.0195110

**Published:** 2018-04-25

**Authors:** Yerali Gandica, Marco Valerio Geraci, Sophie Béreau, Jean-Yves Gnabo

**Affiliations:** 1 CeReFiM (DeFiPP), Université de Namur, Namur, Belgium; 2 Namur Center for Complex Systems - naXys, Université de Namur, Namur, Belgium; 3 ECARES, Université libre de Bruxelles, Brussels, Belgium; 4 CORE, Université catholique de Louvain, Louvain-la-Neuve, Belgium; Rutgers The State University of New Jersey, UNITED STATES

## Abstract

Drawing on recent contributions inferring financial interconnectedness from market data, our paper provides new insights on the evolution of the US financial industry over a long period of time by using several tools coming from network science. Relying on a Time-Varying Parameter Vector AutoRegressive (TVP-VAR) approach on stock market returns to retrieve unobserved directed links among financial institutions, we reconstruct a fully dynamic network in the sense that connections are let to evolve through time. The financial system analysed consists of a large set of 155 financial institutions that are all the banks, broker-dealers, insurance and real estate companies listed in the Standard & Poors’ 500 index over the 1993–2014 period. Looking alternatively at the individual, then sector-, community- and system-wide levels, we show that network sciences’ tools are able to support well-known features of the financial markets such as the dramatic fall of connectivity following Lehman Brothers’ collapse. More importantly, by means of less traditional metrics, such as sectoral interface or measurements based on contagion processes, our results document the co-existence of both fragmentation and integration phases between firms independently from the sectors they belong to, and doing so, question the relevance of existing macroprudential surveillance frameworks which have been mostly developed on a sectoral basis. Overall, our results improve our understanding of the US financial landscape and may have important implications for risk monitoring as well as macroprudential policy design.

## Introduction

The strong interdependence among financial institutions has been emphasised in numerous academic contributions, mainly after the 2007–2008 worldwide financial crisis, leading to a gradual shift from a micro to a macroprudential approach to financial stability. According to the Financial Stability Board (FSB) and the Basel Committee for Banking Supervision (BCBS), financial interconnectedness, defined as the network of contractual obligations which can potentially channel financial distress [1], is now considered as a major determinant of systemic risk along with cross-jurisdictional activity, size, substitutability and financial institution infrastructure, and complexity. Yet, the progress made to exploit the potential of this system-wide framework is still limited compared to other fields, such as physics, neurology or biology to quote only a few, in which network and complex systems’ representations have been deeply rooted for decades [2, 3]. Given the importance of financial stability on economic growth and welfare, it appears critical to pursue the effort in this direction and to accelerate the “transfer of knowledge” from other fields to expend our understanding of financial systems.

Against this background, the aim of this paper is twofold. On the one hand, it brings up-to-date knowledge from network science to economic and financial systems. More specifically, we use a large set of tools devoted to the analysis of network structures in order to improve our understanding of the US financial industry over a long time span. By doing so, we are able to identify phases of integration and fragmentation in the industry, both within as well as across financial sectors. As such, our results can document among others the debate regarding the relevance of sector-based metrics for macroprudential surveillance. For instance, in 2011, the Bank of New York Mellon, State Street Corporation, and Northern Trust Corporation (collectively “Specialized Custody Banks”) had addressed the following concern to the Basel Committee on Banking Supervision: “In its assessment of interconnectedness category as used [for measuring systemic importance of banks], we recommend that the Proposal better define ‘Financial Institution’ in designating the assets and liabilities to be included in these indicators. For example, it is unclear the extent to which the definition of ‘Financial Institution’ includes collective investment vehicles such as mutual funds, collateral investment pools, or other types of private funds and investment vehicles.”. On the other hand, the specificities of our dataset, as detailed below, and in particular the observation of a highly dynamic system over a long period of time, are intended to provide new insights to network science.

One of the greatest difficulties faced by researchers when analysing financial networks lies in the lack of reliable and comprehensive datasets on the “physical” relationships between institutions. To deal with this issue, a now common approach in the literature consists of making use of information embedded in market-based data as done in [4, 5]. In a nutshell, financial institutions represent the nodes of the network with the linkages reflecting the relative influences between pairs of firms, which corresponds for instance to how much stress for an institution as materialised by severe losses, is transmitted to another institution. The identification of those links is based on the statistical measurement (be it correlation or other forms of causal measure, for instance) of temporal dependences between one or several observable characteristics associated to the nodes, such as their stock market returns. Relying on this approach to form pairwise connections instead of observed physical contracts or transactions between institutions implies that the information propagating over the system, far from being innocuous, strongly affects all the nodes in the path. In other words, the links of our system reflect “effective” transmission channels of financial distress (i.e. based on changes in the firms asset value) as opposed to “potential” channels stemming from physical connections, such as cross-lending or common portfolio holdings. In this paper, we follow a market-based approach to retrieve the network representation of the financial system whose characteristics will then be studied by means of several network metrics successively considering individual, then sector-, community- and finally system-wide analyses. More specifically, we apply the “causal” version of this approach in which statistical dependences are assessed with time lags to recover the directionality of the relationship and in turn, separate influencer from receiver nodes. Another important aspect of our system is that nodes are institutions belonging to four different financial sectors, i.e. banks, broker-dealers, insurance companies and real estate companies. As a consequence, we are able to study whether the level of potential disruptions caused by any propagation mechanisms may depend, not only on the structural position of the nodes in the network (i.e. their level of criticality), but also on the sectors those nodes belong to. Importantly, our network is fully dynamic in the sense that it is analysed over successive periods with potential changes within its structure over time. Last but not least, we go beyond sector-based categories to identify sub-groups of institutions by using a more agnostic approach consisting in applying a community detection algorithm which allows to recover “data-driven” groups of highly connected financial institutions.

Following [6], the linkages between financial institutions are retrieved from stock market prices by means of Bayesian Time-Varying VAR framework in the vein of the one developed by [7] for macroeconomic data. One of the main advantages of this methodology is that it allows to generate temporal networks in a widely spaced period of 20 years, where connections are evolving gradually through time. Another powerful characteristic is that it does not use the common rolling window approach, where causal relationships are estimated over successive sub-samples which may lead to several empirical problems that may blur the identification of real linkages between nodes. Instead, statistical inference is established by taking into account the whole temporal spectrum of the data, therefore overcoming some limitations of rolling-window approaches, approaches, regarding sensitivity to window sizes and outliers. This methodology is applied to a large dataset that can reasonably be considered as an adequate representation of the US financial system over two decades as it embeds all the financial firms listed in the S&P 500 index, belonging to the four previously mentioned sectors. Overall, it includes 155 financial institutions for which we observe monthly stock prices from April 1993 to November 2014. Working at a relatively low frequency allows avoidance of potential problems due to stock market noise and eases the identification of causal linkages. This analysis is performed at the system wide, sectoral and community levels. For the former approach, we apply the so-called “Louvain method” on our networks at each period considering undirected edges. By doing so, we recover a set of communities within which financial institutions could influence each others. The “Louvain method” has been widely used in the network science literature to isolate groupes of highly connected nodes.

Equipped with this dynamic representation of the US financial industry, our main goal is then to describe its characteristics with a set of metrics taken from network science. Accordingly, we do not intend here to provide an in-depth analysis of the impact of network topology on market stability, nor do we document the driving factors of network formation. Such avenues in a context in which the dynamic nature of financial systems has been properly featured are left for future research. We believe, however, that this contribution provides important insights to complement existing studies that use network representations to analyse the financial industry. Among the most related contributions, we can quote [4, 5]. In their pioneering works, they propose a way to overcome the absence of comprehensive data on “physical” relationships between financial institutions, by inferring the network from stock market data. Applying Granger-causality tests on bivariate models [4] or more sophisticated Vector Autoregressive model and variance decomposition [5], those works provided the first set of empirical evidence regarding the structure of interconnectedness for the financial industry, including banks, insurance companies or investment funds. One of the main caveats of this generation of studies lies in the way the temporal nature of financial networks was dealt with as they did rely on successive snapshots of the industry at different time periods by means of rolling windows regressions. Recently, there have been a few studies among which [6, 8] some have developed methodological approaches specifically designed to better tackle the time varying dimension of the financial system and infer fully dynamic networks. However, these contributions do not exploit in full the set of tools offered by network science to document salient patterns, relying on degree centrality measures. We propose to fill the existing gap in this contribution by addressing these two caveats together.

By doing so, we more specifically contribute to the literature by shedding a new light on the following issues: (i) the influence/vulnerability of financial institutions at the individual and at the sector level, and (ii) the fragmentation/integration of the whole financial industry. To that aim, we concentrate on six specific aspects of the network: (i) degree centrality, (ii) community structure, (iii) component structure, (iv) sector interface, (v) Katz centrality and M-reach centrality (contagion process), (vi) “top” institution behavior. This latter measure is inspired by the one developed in [9]. It is simple to implement and enables easy comparison of the level of connectedness of the different sectors when considering only the contribution of the most connected institutions, that we call “top” institutions.

Our results document 20 years of the evolution of US financial industry. The critical role played by the banking and the real estate sectors in the successive episodes of financial turmoil appears clearly, the former emerging as a major transmitter of risk in the industry, while the latter appears as the main absorber. The insurance companies also appear as central because of both their exposure to the rest of the system and their effect on other institutions. The role of broker-dealers conversely has been more moderated than the three other industries all over the sample. Our results also provide important evidence regarding the co-existence of both fragmentation phases within the financial industry along with overall increasing integration among firms, in whatever the sector they belong, which, as a result, questions the accuracy of sector-based macroprudential frameworks, the current supervision system being articulated around Basel III for banks, Solvency II for insurance companies and the Markets in Financial Instruments Directive 2004/39/EC (known as “MiFID”) for European investment funds for instance. From a network science perspective, our results illustrate the relevance of the traditional tools developed in this field for analysing “spillover-based” financial networks. Specifically, it shows that relevant information can be retrieved from financial data with centrality measures at the node- and sector-levels. It provides evidence that specific group-detection approaches such as community structure or component structure detection algorithms are of interest to create “agnostic” categories of financial institutions in addition to sector-based groups. It documents the quasi equivalence on financial data of alternative measures such as contagion process and the Katz centrality. Note that for this specific application, we set the number of steps for the contagion process (M-reach centrality) to two, and the damp parameter to 0.625 for the Katz centrality. Lastly, we confirm the interest of extracting information from top values of centrality measures as done among others in [9] in the context of Wikipedia page edition.

The remainder of the paper is the following. We describe in the second section the construction of the network. The third section discusses the results. We start with an analysis of the network’s main characteristics at the sectoral, community and component levels. Then, we examine sectoral interface and eventually discuss tools related to contagion modelling. Finally, the fourth section concludes.

## Methods

To represent the interdependencies at stake in the financial system, we follow [6] who propose a framework based on time-varying parameter vector autoregressions, as in [7, 10] to recover a network of financial spillovers—or causality-based network—that is entirely dynamic. In their framework, financial institutions represent nodes in a directed network. Whereas spillovers, measured as temporal dependence between the stock price returns of the financial institutions, represent the directed edges of the network. Temporal dependence between stock returns is measured according to the following vector autoregression:

$$\begin{array}{c} R_{t}=c_{t}+B_{t}R_{t-1}+u_{t} \end{array}$$ (1)

where $R_{t}={\left[r_{1t},...,r_{Nt}\right]}^{{}^{\prime }}$ is the vector of the stock returns of the *N* financial institutions in the network. *c*_*t*_ is the time-varying intercept, whereas *B*_*t*_ is a *N*x*N* matrix of time-varying autoregressive coefficients, which determines the temporal dependence between the stock returns and therefore the directed spillovers between the financial institutions.

Precisely, a directional edge is drawn at period *t* from *i* to *j*, if the *ji* element of *B*_*t*_, $B_{t}^{\left(ji\right)}$, is significantly different from zero. The framework parallels the classic time-invariant approach of recovering financial spillover networks using Granger causality, see e.g., [4].

Finally, the errors, *u*_*t*_, are assumed to be normally distributed, with mean zero and variance-covariance matrix Σ (see Section A in S1 Appendix of the Supporting Information). The original model of [6] allows for heteroskedasticity and fat-tailed errors. Here, however, we adopt a simpler approach and standardize (so to have unit variance) the returns, *r*_1*t*_, …, *r*_*Nt*_, in a previous step by using a GARCH(1, 1) model to estimate the time-varying volatility.

The model in Eq 1 can be re-written in a compact-form, as

$$\begin{array}{c} R_{t}=X_{t}^{{}^{\prime }}\theta_{t}+u_{t}, \end{array}$$ (2)

where *X*_*t*_ = *I*_*N*_ ⊗ [1, *R*_*t*−1_] and *I*_*N*_ is a *N*x*N* identity matrix. ⊗ is the Kronecker product, and *θ*_*t*_ is a vector with the stacked elements of *c*_*t*_ and *B*_*t*_.

The time-varying parameters are then assumed to evolve according to a random walk:

$$\begin{array}{c} \theta_{t}=\theta_{t-1}+\nu_{t}, \end{array}$$ (3)

where *ν*_*t*_ ∼ *N*(0, *Q*).

This assumption allows the time-parameters, *c*_*t*_ and *B*_*t*_ to evolve flexibly over time and to allow the data to speak by itself. The amount of time-variation, is governed by the variance of the errors, *Q*, which is estimated along with the other parameters of the model, *θ*_*t*_ and Σ.

Finally, in order to determine the existence of a link from *i* to *j*, at a given time period t, we test the following null hypothesis:

$$\begin{array}{c} H_{0,t}:B_{t}^{\left(ji\right)}=0\;\forall j\neq i \end{array}$$ (4)

The model outlined in Eqs 1, 2 and 3 is estimated using Bayesian techniques following [11]. Then, the hypothesis given by Eq 3, is tested using Bayesian inference. Specifically, we use Bayes Factor, which gives the odds in favor of the null hypothesis against the alternative hypothesis, $H_{1,t}:B_{t}^{\left(ji\right)}\neq 0$, without assuming that the null hypothesis is true. Bayes Factor is estimated following [12]. Once we retrieve Bayes Factor, we look at the implied probability that is true. Note that if ${\hat{K}}_{t}^{ji}$ is Bayes Factor for $H_{0,t}^{\left(ji\right)}$, then the implied probability is just ${\hat{K}}_{t}^{ji}/\left(1+{\hat{K}}_{t}^{ji}\right)$.

We use the implied probability to retrieve the network at different cut-off points. Effectively, the cut-off is a filtering mechanism and a higher cut-off leads to a more dense network with more links. The stability of our analysis is assessed by varying the cutoff levels of the statistical test used for detecting the links. In most of the figures throughout the study, the four following cutoffs are considered to be: 5%, 7%, 10% and 15%.

The prior distributions assumed for the parameters to retrieve the Bayesian estimates, are given in Section A of S1 Appendix. The posterior distribution algorithm, which, for the time-varying parameters, uses the Kalman filter and smoother as per Carter and Kohn (1997), is given in Section B of S1 Appendix.

We applied the model to all financial institutions among banks, insurers and real estate companies (SEC codes 6000 to 6799) that were components of the S&P 500 between January 1990 and December 2014. For these companies we collected the stock price at monthly close from Thomson Reuters Eikon over the same time period. Initially the sample contained 182 firms but was reduced to 155 after restricting our analysis to stocks with at least 36 monthly observations. As mentioned, all returns were standardized using a GARCH(1, 1) model, to account for heteroskedasticity, prior to applying the time-varying framework highlighted above.

## Results

### Sector-based behavior

In this section, we provide an overview of the network. The number of nodes in the system as well as centrality measurements are displayed by sector.

**Fig 1:**
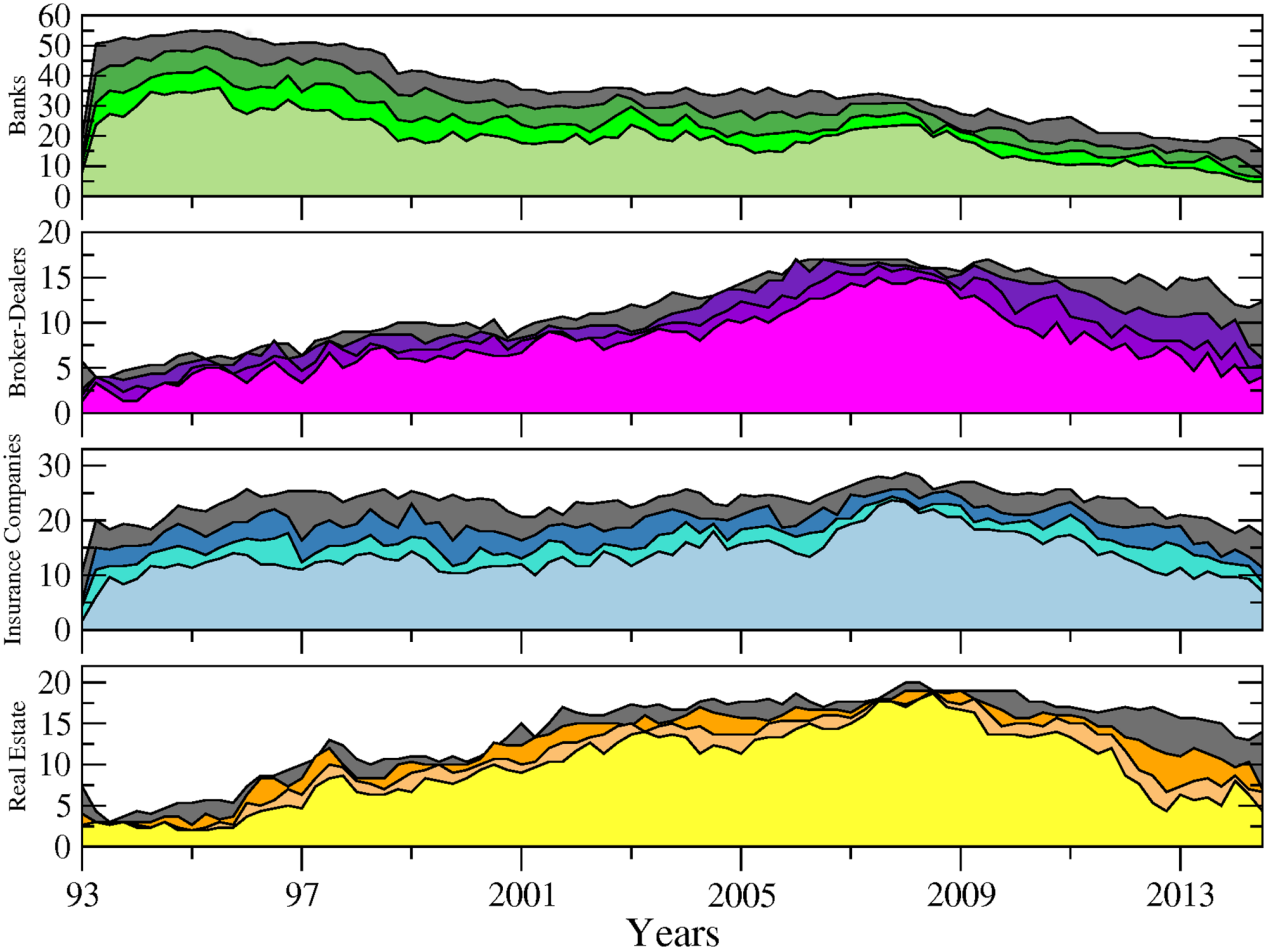
Number of connected nodes per financial sector (i.e. sub-networks) from April 1993 to November 2014. From top to bottom: banks (green), broker-dealers (purple), insurance companies (blue) and real estate companies (yellow). The different tonalities in each plot are related to the sensibility parameter (i.e. test’s cutoff levels for detecting significant links) set to 5% (softest tonality), 7%, 10% and 15% (dark grey) respectively.

**Fig 2:**
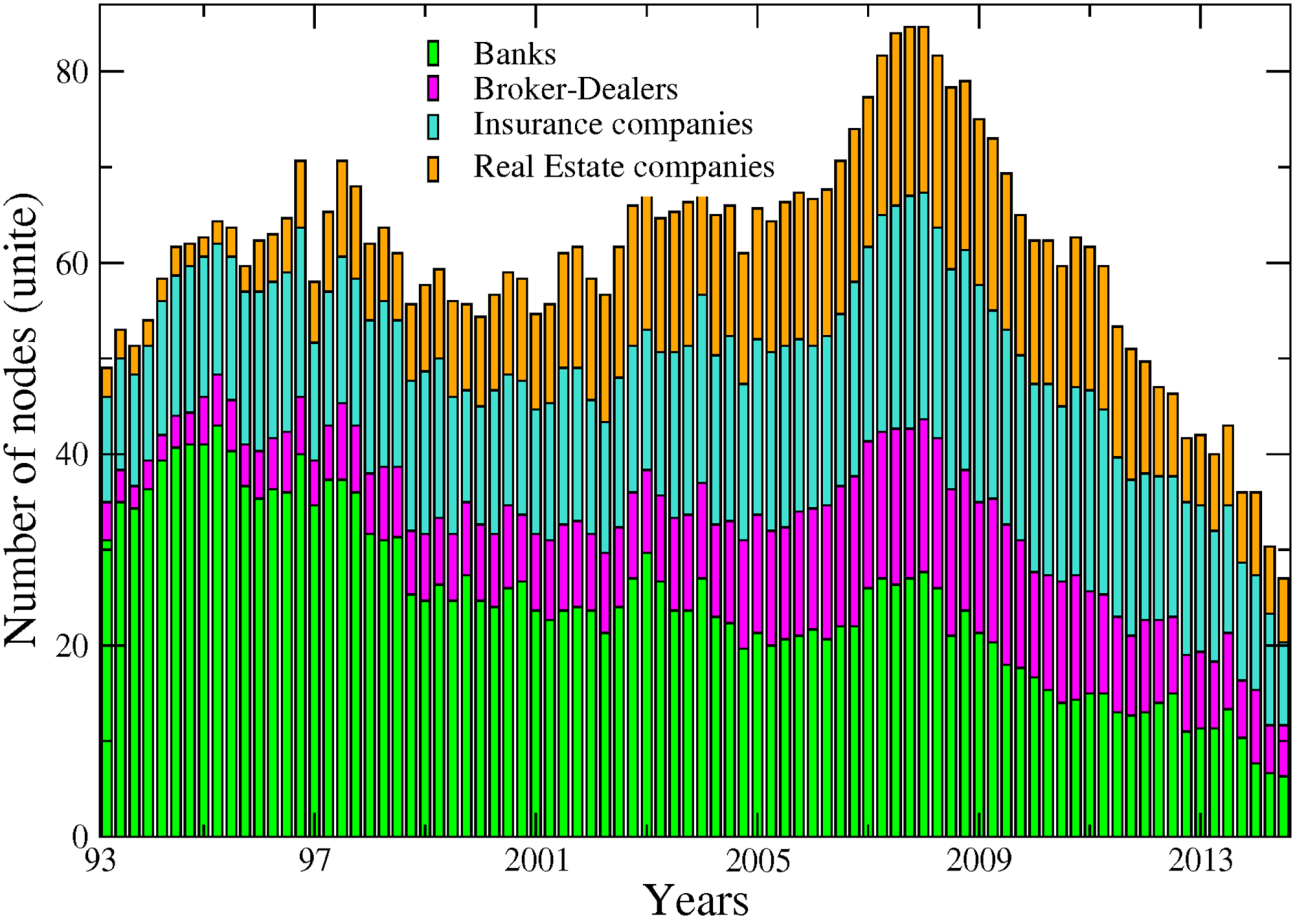
Each bar size represents the number of connected institutions within our four sectors, from April 1993 to November 2014. Banks (green), broker-dealers (purple), insurance companies (blue) and real estate companies (yellow). Values are displayed at quarterly frequency to ease the visualisation. The sensibility parameter has been fixed to 10%.

Fig 1 shows the number of connected nodes comprising our network across time among the different sectors mentioned above. We can notice, from a first visual inspection, that the main patterns characterising the evolution within each sector are not sensitive to the choice of the cutoff level used to detect the links. Comparing now the series across sectors, Fig 1 exhibits contrasting dynamics. In particular, while the number of connected banks has steadily decreased since 1995, insurance companies and real estate companies experienced two successive phases characterised by an upward trend until late 2008 followed by a reversal. The number of broker-dealers overall displays more stability. It is worth recalling at this stage that as our connections are built on causal relationships among asset prices; an institution identified as not connected to the rest of the network can to some extend be physically related to other institutions. However, such relationship does not materialise into spillover effects. Fig 2 completes the picture by adding up the number of nodes across sectors. Note that for the sake of clarity, we will often show across the paper the results related to the sole 10% cutoff level, figure for other significance cutoff levels being available upon request. We can more clearly observe from there the various phases at the system-wide level. From 1993 to 1997, the number of connected nodes sharply increased, being mainly driven by the banking sector. The trend reversed then until 2001, reaching a total of 55 connected nodes from 70 in 1997. From 2001 to 2008, the upward trend resumes to attain the highest level of the whole sample by peaking at more than 80 nodes. Interestingly, as noted in Fig 1, the number of banks decreased over that period. Hence, our data reveals the presence of a shift among the set of large connected institutions from the US financial industry in the run-up to the financial crisis with insurance companies as well as real estate companies, and to a lower extend broker-dealers, gaining in importance within the system. Eventually, the size of the network dramatically shrunk after 2008, reaching its lowest level at around 35 connected nodes—in our case the number of institutions influencing or being influenced by the rest of the system—institutions in late 2013. The four sectors were almost equally affected by this drop. Such a result implies that many institutions became isolated or weakly connected to the rest of the system. It is worth reminding that our identification strategy for the links is based on statistical tests. Therefore, the absence of a link between pairs means that we do not have enough evidence, or, said differently, the evidence is too weak in the data to reject that institutions could be independent.

From this first inspection, we can notice that the dates of the successive turning points correspond to well-known financial events such as the Asian crisis in late 1997, the burst of the so-called “dot.com” bubble in 2001 as well as the 2007–2009 financial crisis.

**Fig 3:**
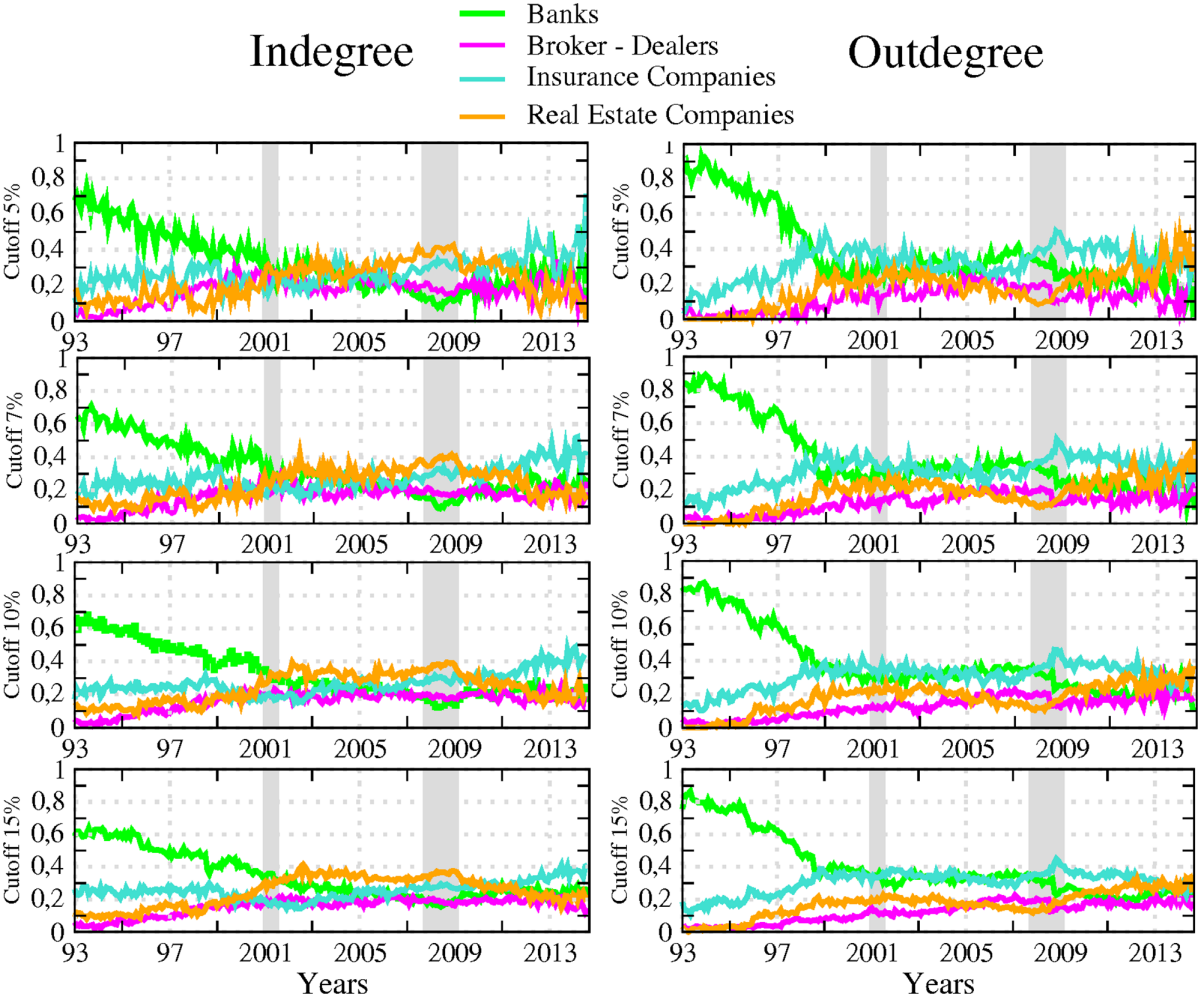
The left (right) panel displays the relative in-degree (out-degree) per sector, from April 1993 to November 2014. Number of incoming (outgoing) links of all the nodes attached to a sector, divided by the total number of links—banks (green), broker-dealers (purple), insurance companies (blue) and real estate companies (yellow). The results are reported for different values of the sensitivity parameter (i.e. test cutoff levels for detecting significant links). The transparent grey corresponds to US recessions as defined by the NBER.

Next, we adopt another perspective by considering directed linkages between the set of institutions constituting our networks. Fig 3 reports the share of incoming (in-degree) and outgoing (out-degree) connections per sector, the share reported for a sector being computed as the ratio between (i) the total number of incoming (outgoing) links attached to the nodes belonging to this sector, that is the in- (out-) degree associated to those nodes, and (ii) the total number of links in the system. Because our directed links depict causal relationship between stock market returns in the sense that they differentiate the transmitter from the receiver of the financial stress, the in-degree allows measurement of how many institutions (from the whole sample) are affecting that sector whereas the out-degree accounts for how many of them are being affected by that sector. In accordance, the former measure characterises the sector vulnerability and the latter its influence. To ease temporal comparisons, we propose a slightly modified version of the raw in- and out-degree centrality measures as we normalise all the values by the total number of links in the system at each given point in time. This measure can be analyzed in level or in variations to visualize more easily periods of acceleration or deceleration in network evolution. For the sake of simplicity, we keep the analysis in level in what follows. A visual inspection of Fig 3 shows a first notable feature for out-degrees: the banking sector experienced more pronounced changes than other sectors, especially in the 90s. This observation is in line with previous discussions in the literature about the large-scale reshaping of the banking industry amid increased competition, consolidation, and efficiency gains. As described in [11], banks embraced the new approach to client-based universal banking during this period, leading to a merger wave in the US banking sector. This change affected the number of banks in the market but also deeply affected their functioning. Our data indicates that it went along a diminution of the influence of the whole sector on the system.

A second notable feature is the occurrence of a shift point at the beginning of 1999. From this date onwards, the drop in the banking sector’s share of outgoing links more or less stopped, to remain stable at around 30% and 40%. By contrast, the insurance companies and, to a lesser extend, real estate firms, followed an opposite pattern. Both sectors experienced an increase in their influence until the late 90s before stabilising around 30% and 35%. From early 2000, the outgoing links of the four sectors have been kept in close ranges with a slight domination of insurance companies which can be deemed in this respect to be the most influential sector in the system, especially once the 2007–2009 crisis burst out. The time series for incoming links offer a different picture. For any of the four reported figures, the cycle of upward and downward trends is less pronounced than for outgoing links as is the heterogeneity across sectors. Banks are still the most connected during the first years of the sample, exhibiting a ratio of incoming links over total links (relative in-degree) of about 60%–80% as compared to about 10%–30% for other sectors. However, their role as an important receiver of spillovers in the system kept decreasing until 2010 before slightly bouncing back. This result complements the well-known dramatic fall in the number of US banks failing over the 90s as reported by the Federal Deposit Insurance Corporation (see https://www.fdic.gov/bank/analytical/banking/2006jan/article2/fig5.htm).

Another interesting result lies in the central role played by the real estate sector which appears as the most exposed sector between the two crises. Hence, from 2001 to 2009, its ratio of incoming links over total links was around 30% compared to 20% on average for the others. This finding means that part of the underlying risk borne by real estate companies before the 2007 crisis was detectable by using this indicator. At this stage however, we should be careful in our conclusions as our measurement of sector-based influence and vulnerability embeds two different effects: a size effect due to the relative importance of the sector in the system and an individual effect corresponding to the connectedness of the nodes populating the sector. Part of the former results for the banking sector in Figs 1 to 3 for instance can be at least partly driven by the fact that many banks have disappeared over the sample. The number of real estate companies and broker-dealers has grown over the years, whereas the number of insurance companies has remained constant. Accounting for these underlying changes in the sample could in turn change the picture.

**Fig 4:**
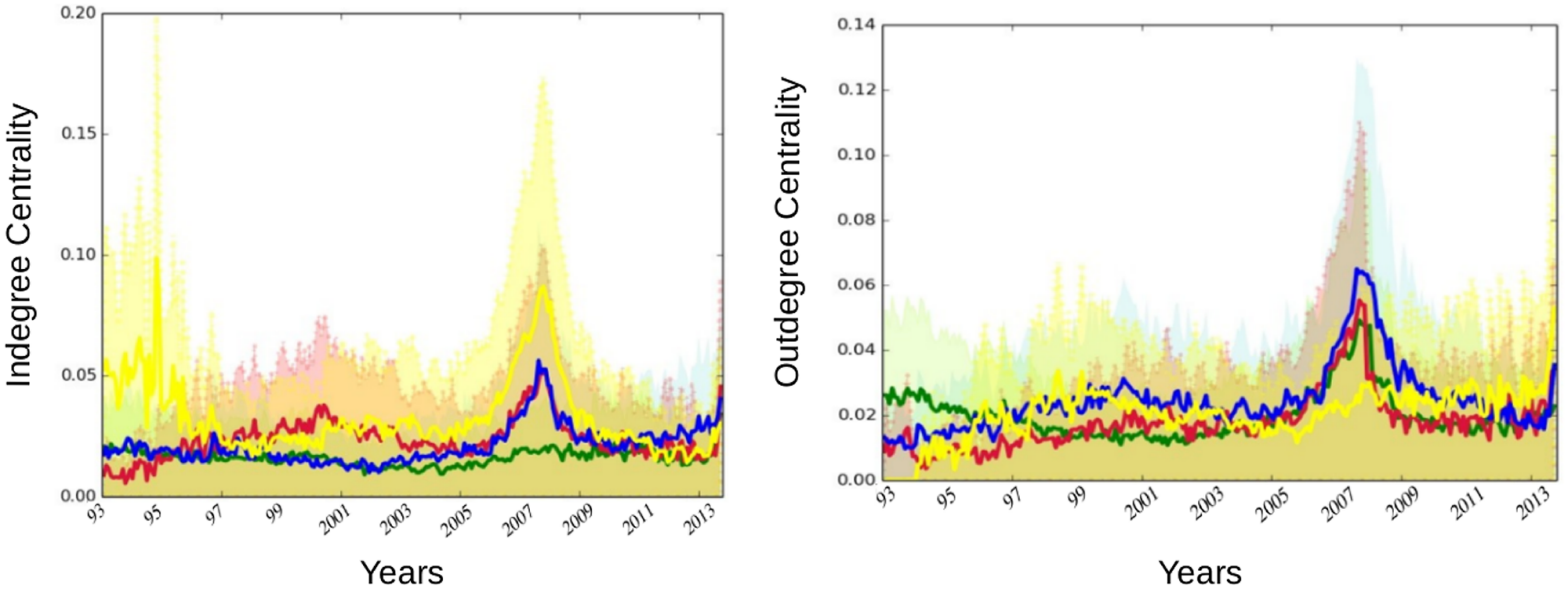
The left (right) panel displays the averaged in-degree (out-degree) centrality, from April 1993 to November 2014. Averaged over sectors—banks (green), broker-dealers (purple), insurance companies (blue) and real estate companies (yellow). The variance of the centrality values is reported in shallow with the same colours.

The next set of measurements builds on those presented in Fig 3, while correcting for the size of each sector. It is computed as the average per sector of outgoing (resp. incoming) links divided by the total number of links. By doing so, we want to abstract any potential size effect (i.e. the fact that some sectors populate the system in greater magnitude) to assess the influential (resp. vulnerable) nature of the components of each sector as opposed to the actual influence (resp. vulnerability) of the sector as a whole. Fig 4 displays the evolution of the in- and out-degree centrality measurements along with their variance. High values of variance are signs of broad dispersion (that is, heterogeneity among firms regarding that specific dimension) within each sector. We can observe that it peaks when both crises occur. A second notable feature is that while the banking sector remains the most influential sector in the early 90s, the picture regarding its vulnerability with respect to the rest of the system changes markedly once we control for the size. Now, it does not appear as different from others. This result illustrates the interest of computing various measurements as they can provide different information. The way to interpret this finding is that banking institutions were not more vulnerable than other financial actors when taken individually. However, because the sector was the largest (i.e. had the highest number of connected actors) in the financial industry at the time, it was the greater receiver of spillovers. The figures regarding relative incoming links (Fig 3) were therefore mainly driven by a size effect. Another point calling for attention is the bell-shape curve observed at the time of the 2007–2009 financial crisis for all the series, with the notable exception of the banking sector relative to incoming links. Such a pattern confirms increased connectedness in the run-up to the crisis and a fragmentation of the US financial network in the aftermath of the Lehman Brothers collapse. If we compare the respective position of each series, our results also confirm that the banking sector was mainly a transmitter of risk rather than a receiver during the crisis, the real estate companies appearing as the main vulnerable element of the system with the highest average of incoming links. Eventually, the figure unveils a novel feature about insurance companies which exhibit the highest level of influence on the rest of the system from 2000 to 2010.

**Fig 5:**
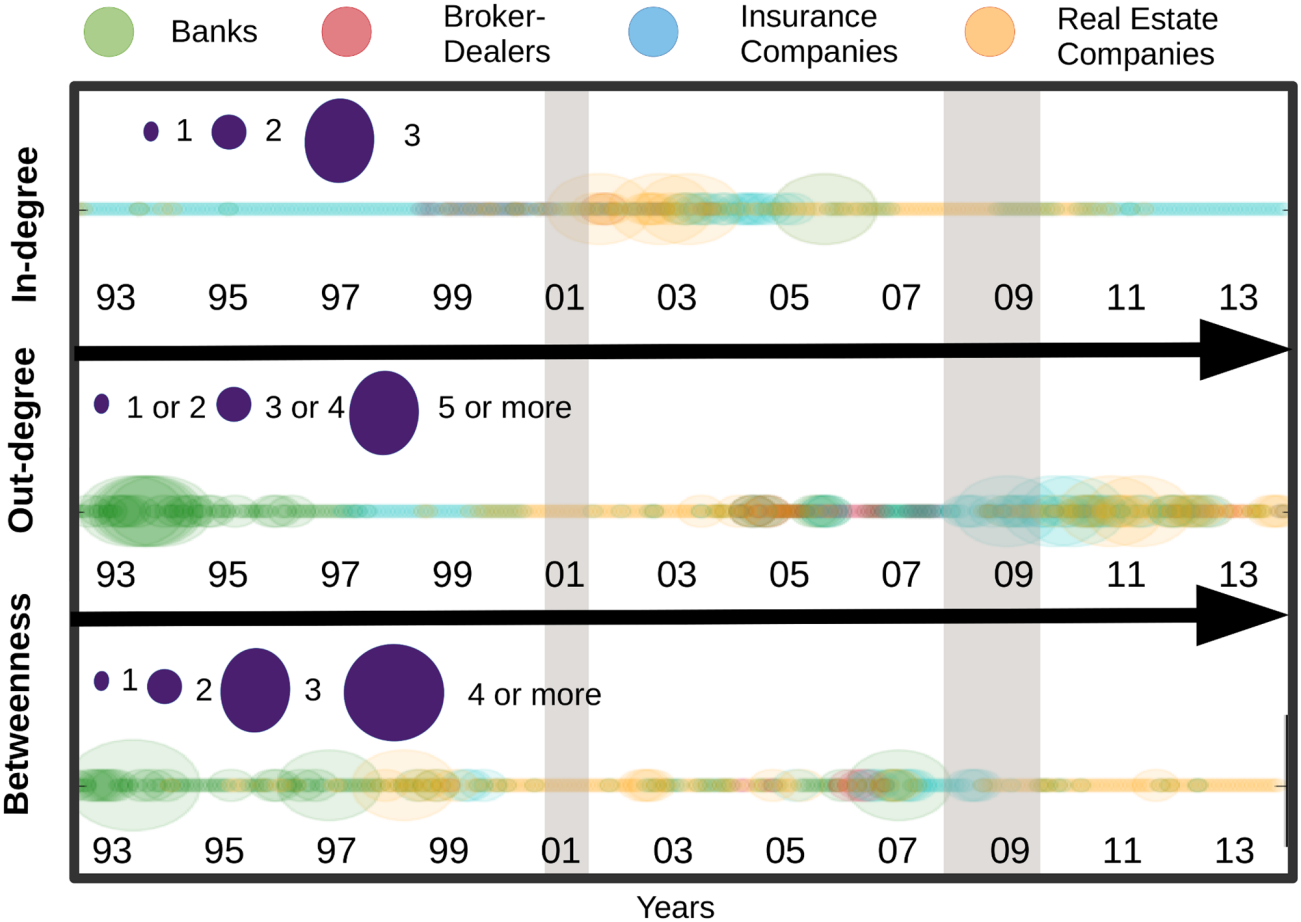
In-degree, out-degree and betweenness top rank sectors, from April 1993 to November 2014. The size of the circles represents the number of institutions with in-degree, out-degree and betweenness among the top 20% highest values at each period.

We now focus on the most extreme cases that we call “top” institutions by considering the concentration of institutions within the top 20% values of the two metrics discussed above. To that aim, we consider for each month the different centrality measurements across institutions. We rank all the values in descending order. We take the first one (i.e. the highest value), then, we create a threshold equal to 80% of the highest value. The range between the highest value and the threshold corresponds to the top 20% highest values. Next, we count the number of institutions per sector falling into the top 20%. The outcome of the procedure measures the level of concentration of institutions among the highest centrality values for a given month. It also indicates whether the most central institution is isolated or part of a group. If the resulting values are low, for instance, it means that one institution stands out above the others in term of centrality. Conversely, if they are high, it means that several institutions potentially belonging to the same sector are important contributors to the connectivity of the system. When centrality is interpreted as a source of risk it means that the risk is not driven by a single institution but by a set of institutions. Fig 5 displays the results for relative in and out-degree centrality along with the traditional betweenness centrality measure. For instance, it shows that among the top 20% highest values of relative outgoing links, three institutions (nodes) were associated to banks in 1994 and three to real estate companies in 2011.

Therefore, just a few institutions stand out in terms of centrality during these two periods and among them we counted several banks in the first years of the sample and several real estate companies at the end. More generally, we observed groups of influential institutions instead of isolated top institution in the early 90s and in the banking sectors before shifting to real estate and insurance companies in the aftermath of the 2007–2009 crisis. Such a feature illustrates how such representation can be of interest for documenting and analysing sector-related patterns versus institution-specific patterns. Turning to relative incoming links, the concentration of the main receivers reached its highest level between the two crises with a dominance of real estate companies and, to a lower extent, insurance companies. The role of real estate companies and banks is in line with previous evidence from Figs 3 and 4. Fig 5 also provides new insights, especially regarding the vulnerability of insurance companies in the years preceding the crisis. Likewise, the figure more clearly points out the concentration of most vulnerable institutions in the run-up to the crisis between 2001 and 2007. The third measurement added to Fig 5 relied on betweenness centrality. Betweenness centrality, in networks representation takes high values for nodes with central location. Being central refers for a node to the number of minimum paths between two other nodes in the network passing through it. It is worth noting, however, that the measure is built on undirected links, making its interpretation difficult in the context of risk propagation analysis. Therefore, its inclusion has to be mainly viewed as a benchmark. Our results show notable discrepancies between the patterns that emerged from in- and out-degree centrality measures and the betweenness centrality, stressing the influence of considering the direction of the links when constructing such metrics. We also detect similarities. In the three cases, for instance, the most pronounced changes over the period covered correspond to well known financial events. More specifically, we uncover a concentration of top institutions outside the crisis periods. This feature is consistent with the idea that financial risk is building up in periods of calm—here the risk corresponds to increased connectedness—and eventually materialised into a sudden collapse of the system before building up again. Such correspondence between the centrality measures and financial events tends to confirm the relevance of top representations for analysing financial networks and systemic risk.

### Components and communities

In the previous sections, we have analysed groups of institutions based on their sectoral classification, which correspond to ex-ante information. In the subsequent section, we now consider an alternative (ex-post) approach according to which institutions sharing strong connections as identified by specific algorithms are included in the same group as done in [13, 14], for instance. More specifically, we consider two different approaches. The first one relies on the identification of specific “components”, that is sets of nodes that are connected by means of at least one link between any pair of them. In our temporal network, several institutions are deemed to belong to the same component if they are connected to at least one of the nodes included in the component structure. Remind that two nodes are directly connected in our context if there exists a statistical dependence between the stock market returns’ time series, associated to that pair, during that month. For this analysis, peer-interdependence in stock market prices is used with a cutoff (test’s cutoff levels for detecting significant links) of 10%.

Given the “soft” character of this measurement, the most useful information we can retrieve from its implementation on our data is concerned with the composition of the small clusters that we identify not to belong to the biggest component. These clusters are made of institutions independent from the global system but still connected in smaller inter-dependent groups. For instance, in 1993 the biggest component included about 50 nodes. Four small components of two or three nodes each could be detected, leaving about 30 nodes unconnected or too weakly connected to be considered as such to the rest.

**Fig 6:**
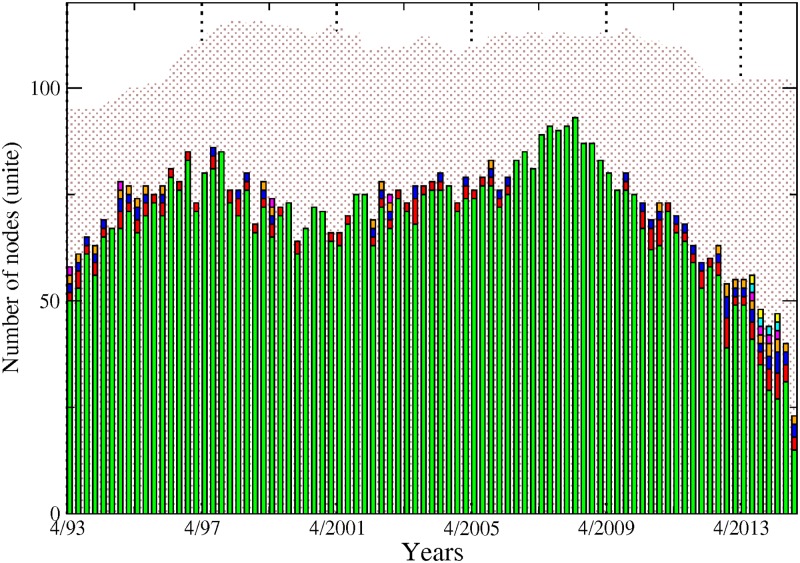
Each bar represents the size of the components, from April 1993 to November 2014. The dotted background depicts the total number of institutions for each month. The green colour is attached to the biggest component, the red colour to the second biggest and so on.

The first notable observation in Fig 6 is the agglomeration of almost all the institutions in the biggest component over the whole sample, denoting global dependence of institutions among financial companies in the US. Yet, the number of non-connected or isolated, institutions is not negligible, as represented in dotted background. In most of the cases, there are also a few institutions that are gathered in very small groups. The 2007–2009 crisis period is particularly interesting. We observe a growing integration of the US industry as materialised by the detection of only one single large component in 2007 and 2008 as opposed to two, three or four small components in the years before. During this period, the largest component reaches its sample peak at 90 (for a total of about 110 nodes at this date). Since then, a fragmentation process has been ongoing until 2014 with both more components and an abrupt and monotonous drop in the total of connected nodes.

**Fig 7:**
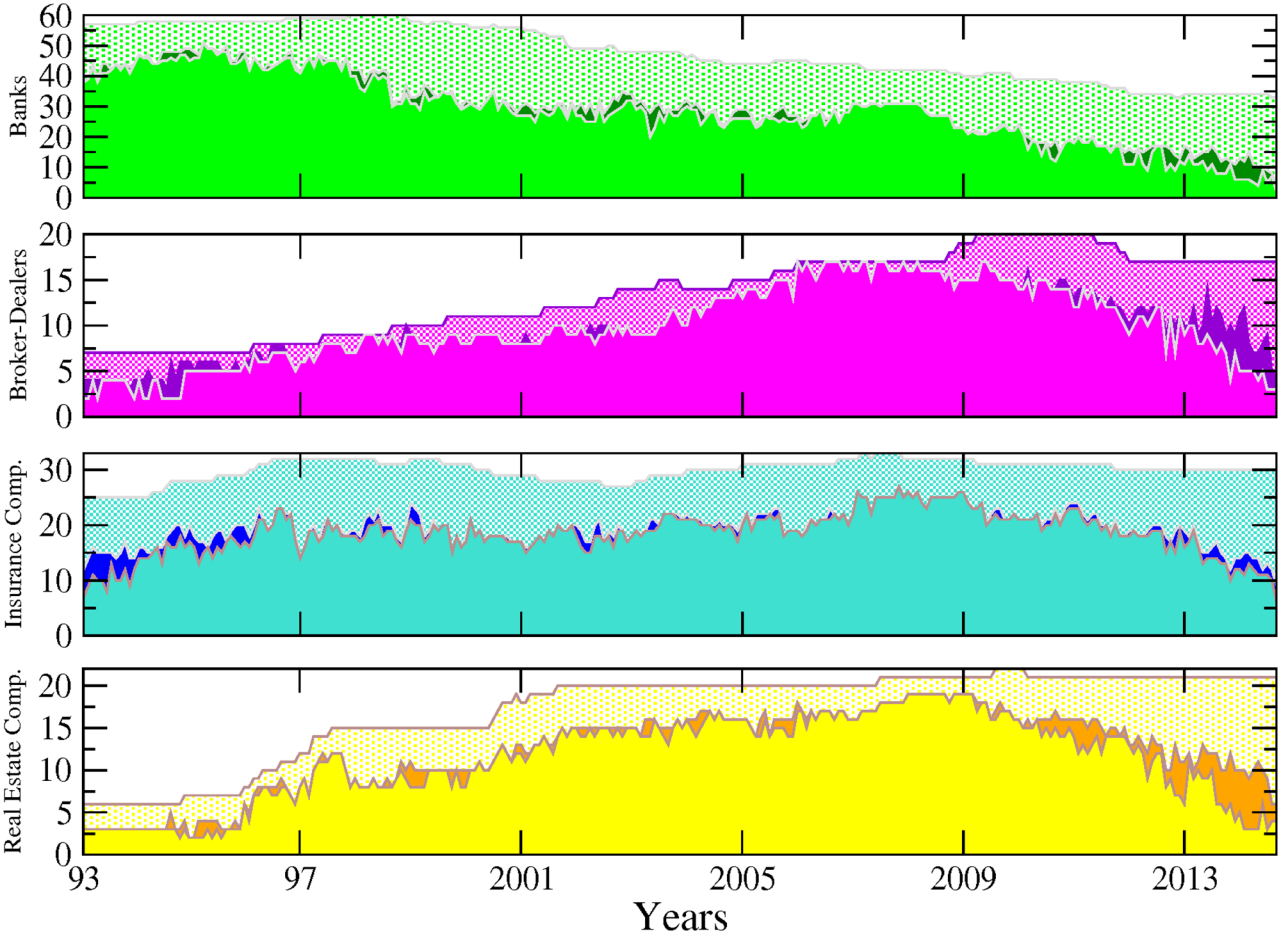
Number of institutions per sector broken down into the number of institutions in the biggest component (plain-light colour), the number of institutions in small components (plain-dark colour) and the number of isolated institutions (dotted-light colour), from April 1993 to November 2014.

Fig 7 provides complementary insight by combining pieces of information on the components and on the sectors. Two features emerge from this exercise. First, the picture provided by the banking sector is contrasted. On the one hand, most of the institutions from that sector are part of the biggest component and only very few of them belong to smaller components. On the other hand, many banks and insurance companies appear isolated. The situation is more balanced for other sectors. Broker-dealers and real estate companies are mainly integrated within the biggest component at the beginning of the sample while a non-negligible part of these institutions move to small components or become isolated in the post-crisis period. A detailed study about the formation (and stability) of the components is out of the scope of the present study, but this analysis might be of interest for future research.

**Fig 8:**
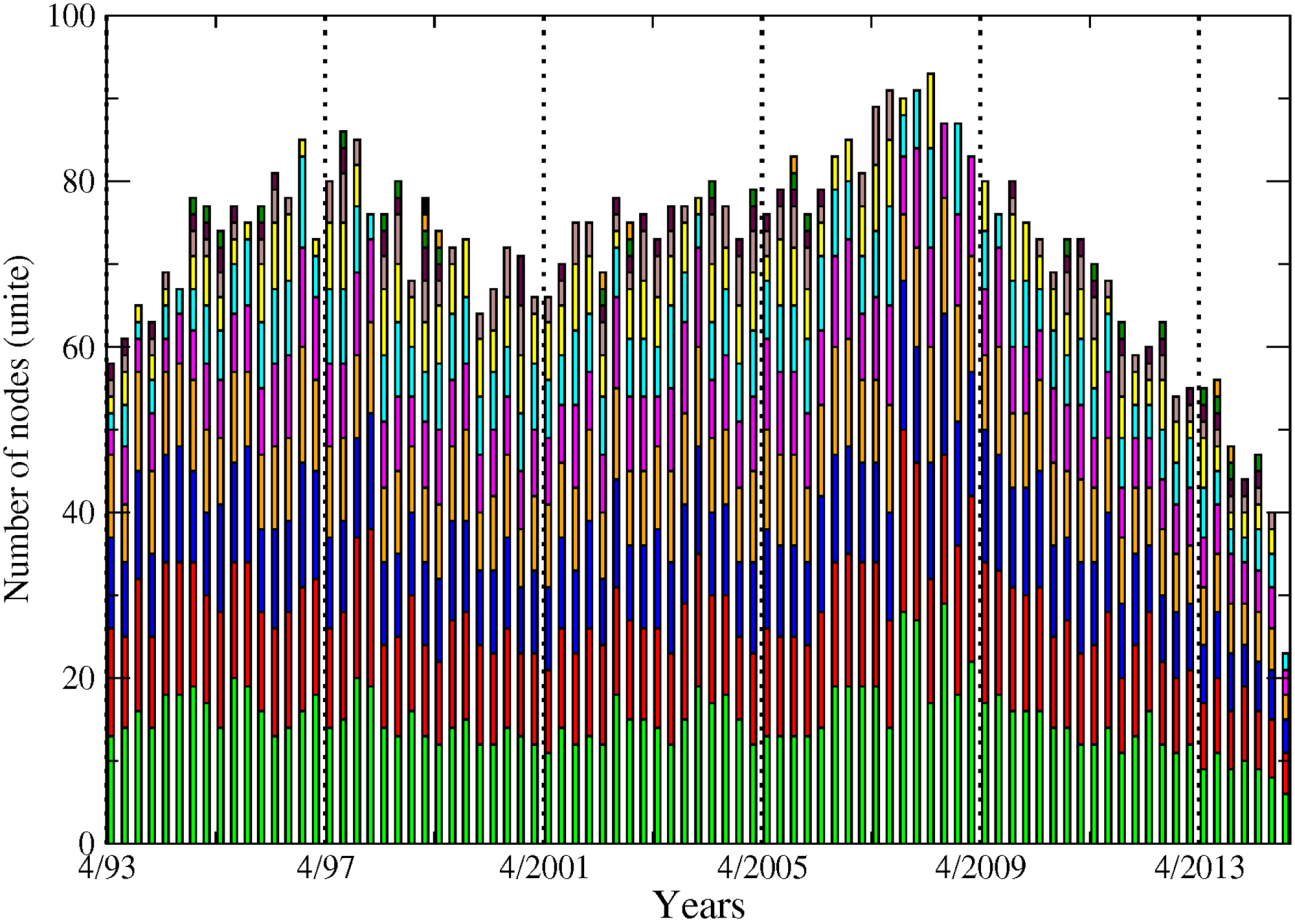
Each bar represents the number of institutions making up each community within the giant component, from April 1993 to November 2014. From bottom to top, the green colour is attached to the biggest community, the red colour to the second biggest and so on.

The classification in components is related to the occurrence of at least one link between pairs of nodes. A more nuanced way to gather the nodes into relevant groups of interacting entities relies on the identification of community structure. A community is a set of nodes more connected between them than with the rest of the system. There exists several algorithms to identify community structures. Our community detection was performed relying on the Louvain algorithm [15]. The goodness of a partition is measured by the modularity. For the undirected version of our networks (where the community detection has been performed), optimising modularity can be interpreted both, as optimising a particular stochastic block model and a particular diffusion process on the networks [16]. The best partition is the one that maximises modularity. In our causality-based networks, institutions grouped into communities means that their market returns display strong temporal dependences. Fig 8 reports the number of institutions constituting communities within the biggest component. We recall that a detailed analysis of the dynamic nature of those communities and their determinants over time is out of the scope of the study. However, we can make some general remarks from this figure, such as the presence of a higher number of communities than the number of sectors, showing that both pieces of information are not redundant. Also, it seems that the community structures exhibit strong stability over time. For instance, we do not observe the same downward slope pattern that was noticed for the number of connected banks in Fig 8. We do observe, nevertheless, a slight peak around the time of the 2007–2009 crisis, characterised by a small decrease in the number of communities and an increase in the number of institutions included in the biggest components—sum of institutions across communities, meaning that communities size expanded during this period. Interestingly, the patterns observable in Fig 8 are very much comparable to those discussed for Fig 6, that is, higher integration during the 2007–2009 crisis and increased fragmentation afterwards, characterised by the emergence of many clusters of institutions.

### Interface

**Fig 9:**
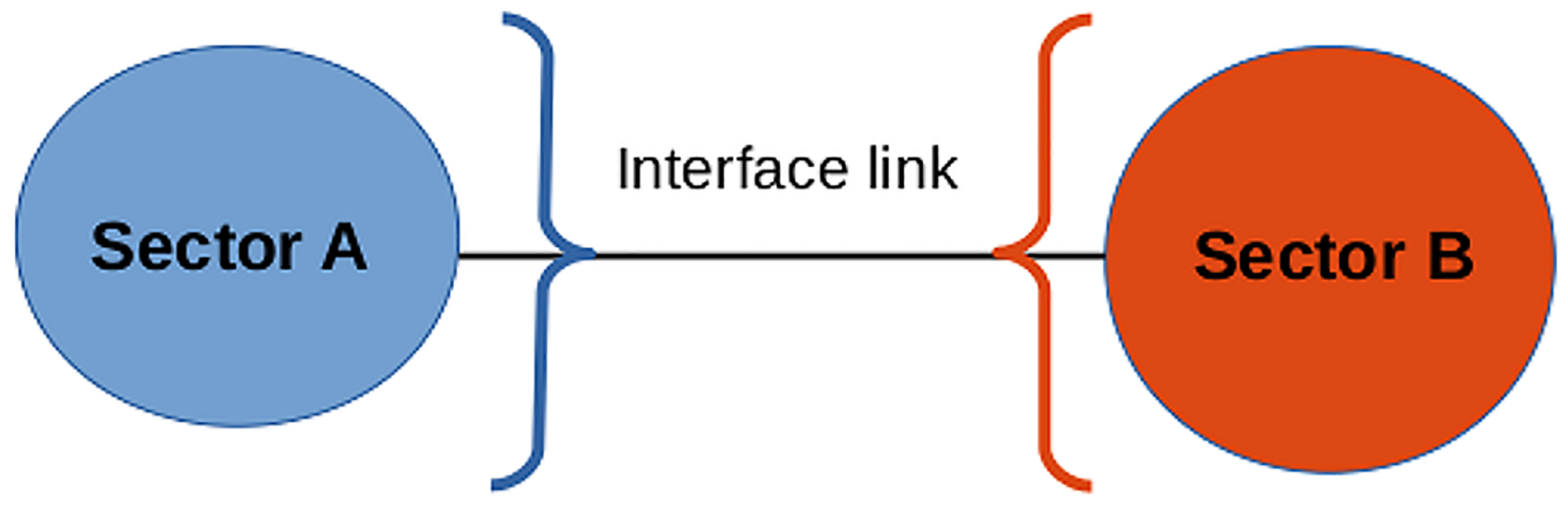
A link is defined as part of the interface if its two connecting nodes belong to different sectors.

**Fig 10:**
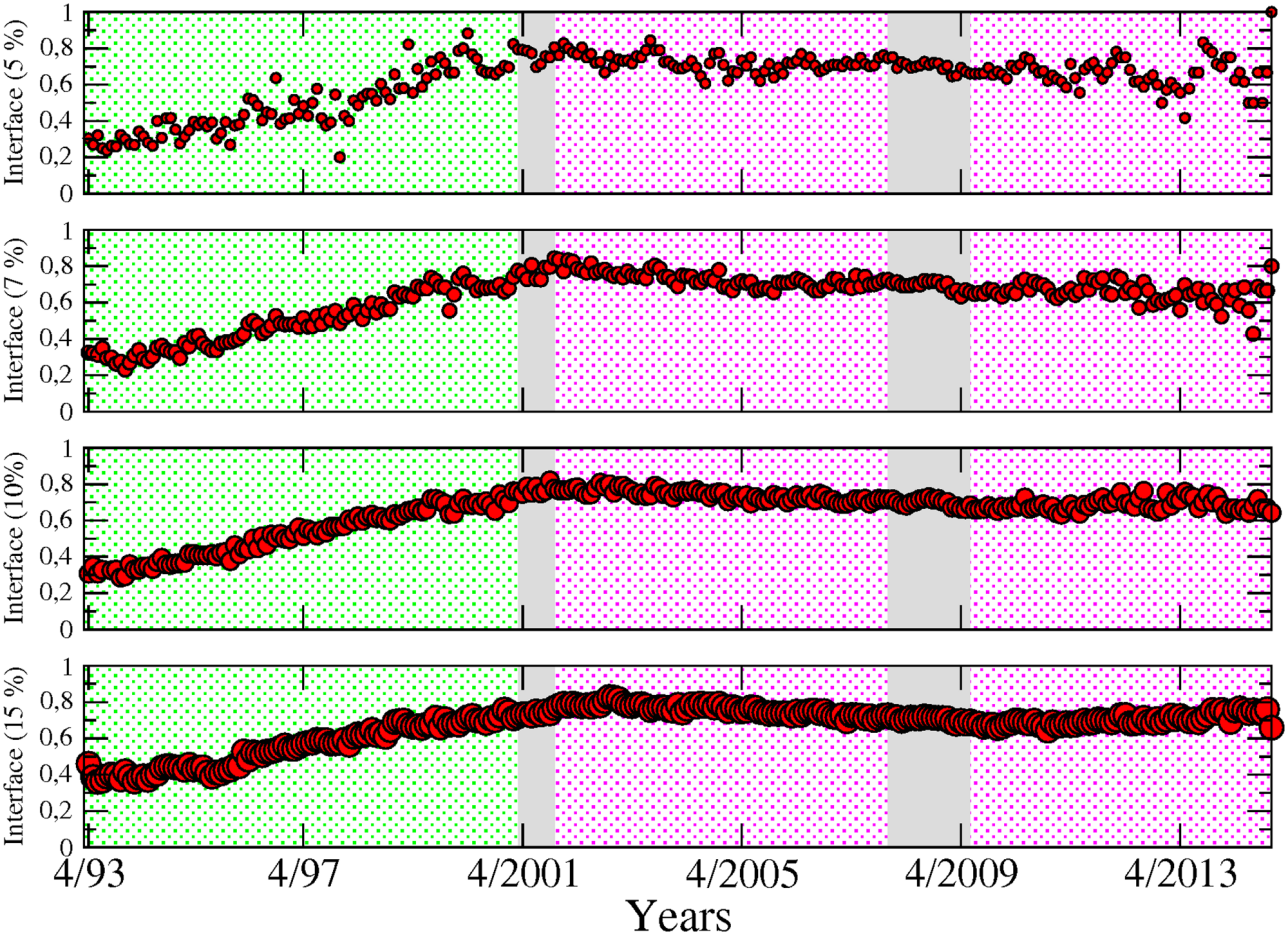
Proportion of sector-interface inside the giant component from April 1993 to November 2014, for different values of the test’s cutoff levels for detecting the significant links. Two different background colours are used to indicate two different phases: in green, a monotonous increasing phase, and in pink, a stable phase around 0.8. It is important to notice that the rise is not a consequence of increments in the number of links, as the values showed are normalised. The measurements are shown for different values of the sensitivity (i.e. cutoff) parameter.

This section aims to complement previous analysis by combining information on institutions’ linkages and their sector-based classification. To that end, we use the notion of “sectoral interface”. We define a link as being part of the interface if it connects two nodes belonging to different sectors (see Fig 9). In Fig 10 we show the proportion of sectoral interface inside the largest component. Two different background colours are used to indicate two successive phases. In green is a monotonous increasing phase, where the proportion of links between different sectors is growing until the first 2001 crisis, starting from around 35% in 1993 to 80%. After this turning point, in pink, the proportion of inter-sectoral links stabilise while keeping fluctuating around 80%. It is important to note that the rising pattern is not a symptom of an inflated number of links in the system, as the values shown are normalised. The measurements are shown for different values of the sensibility parameter (5, 7, 10 and 15%), in order to illustrate the robustness of the results. The pattern that we identify here in sectoral interface illustrates the high level of integration of the financial industry and in particular, the presence of strong connections across sectors that can be traced back long before the occurrence of the 2007–2009 crisis. Also, it shows that the level of integration across sectors was far lower in the 90s, a decade which experienced several large-scale bankruptcy episodes in the financial sector such as the failure of LTCM with less comparable impact on the real economy than the 2000s crises. The transition phase that we have been able to identify echoes previous results in the literature reporting the existence of similar transition phenomena in economics [17, 18].

### M-reach (contagion processes) vs. Katz centralities

We complete our analysis of the financial industry by going beyond direct connections and considering indirect ones. To this end, we use the Katz centrality measure as in [19], along with the contagion process which has been more considered in financial application. Katz centrality is a metric used to understand the importance of each node. It measures the connections of a node with other nodes of the network through both direct and indirect links. The weight attached to each direct and indirect connected nodes is a function of the distance between the two nodes. In accordance, the more indirect the connection—that is, the more intermediate nodes—the higher the distance and the lower the weight. Formally, the distance is featured through a penalising attenuation factor. The Katz centrality is a good alternative to eigenvector centrality for measuring centrality beyond first-degree nodes when the network is sparse or directed.

**Fig 11:**
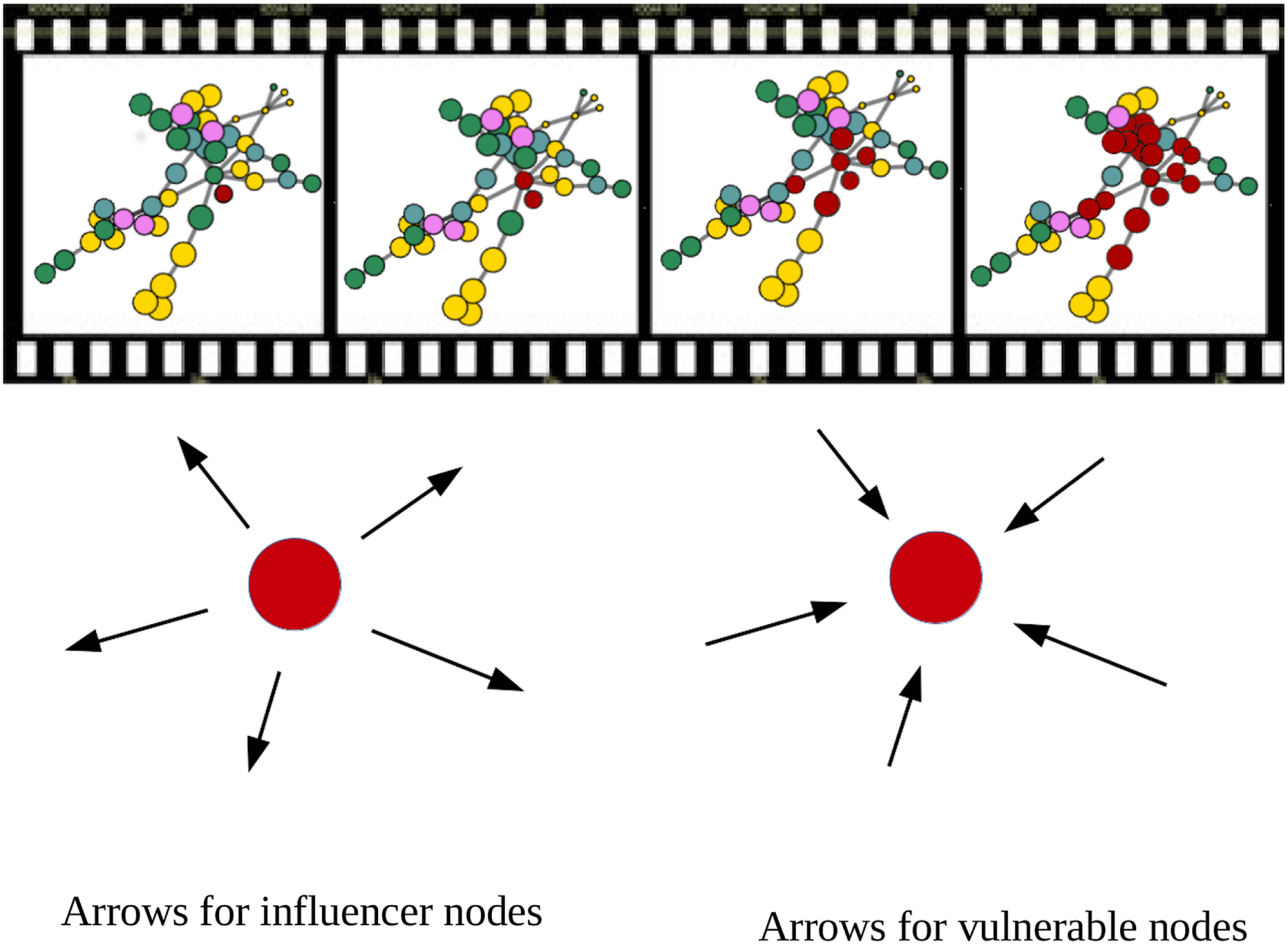
Infection process. Top figure: Infection process in three steps to calculate the biggest influencer and vulnerable nodes. Bottom figure: Direction of the arrows to calculate the influencer institutions (left) and the vulnerable nodes (right).

An alternative and intuitive approach to Katz centrality is achieved by applying percolation theory and computing the M-reach centrality. In the context of outgoing links, it builds on a very similar principal to the Katz centrality measure by identifying how influential each node is in its ability to spread infection or shocks—in our case severe losses—to the rest of the system. In our simple contagion process, we start with an infected node as an initial seed and then we count the number of nodes touched by this infection through outgoing links (see top panel of Fig 11). We can illustrate the algorithm with the following example. Starting from one node with three outgoing links, three nodes are infected in the first step. In the second step, the three infected nodes become diffusers of the disease to their neighbours via their outgoing connections and so on. We stop the procedure at the third iteration and count the total number of infected nodes. Note that the contagion process will highlight the structural role of nodes. For instance, the target node in Fig 11 is weakly connected to the network and with a peripheral role; however, its connectivity to a highly central hub contributes structurally to global connectivity. To detect vulnerable nodes, we apply the same procedure, having initially inverted the direction of all the arrows in the networks (see bottom panel of Fig 11). After three steps, the higher the number of infected nodes, the higher the number of institutions influencing the stock market price of the institution from which we started. The effect of inverting the arrows over the contagion process gives the number of influencer institutions each node is vulnerable to, then characterising its level of fragility.

**Fig 12:**
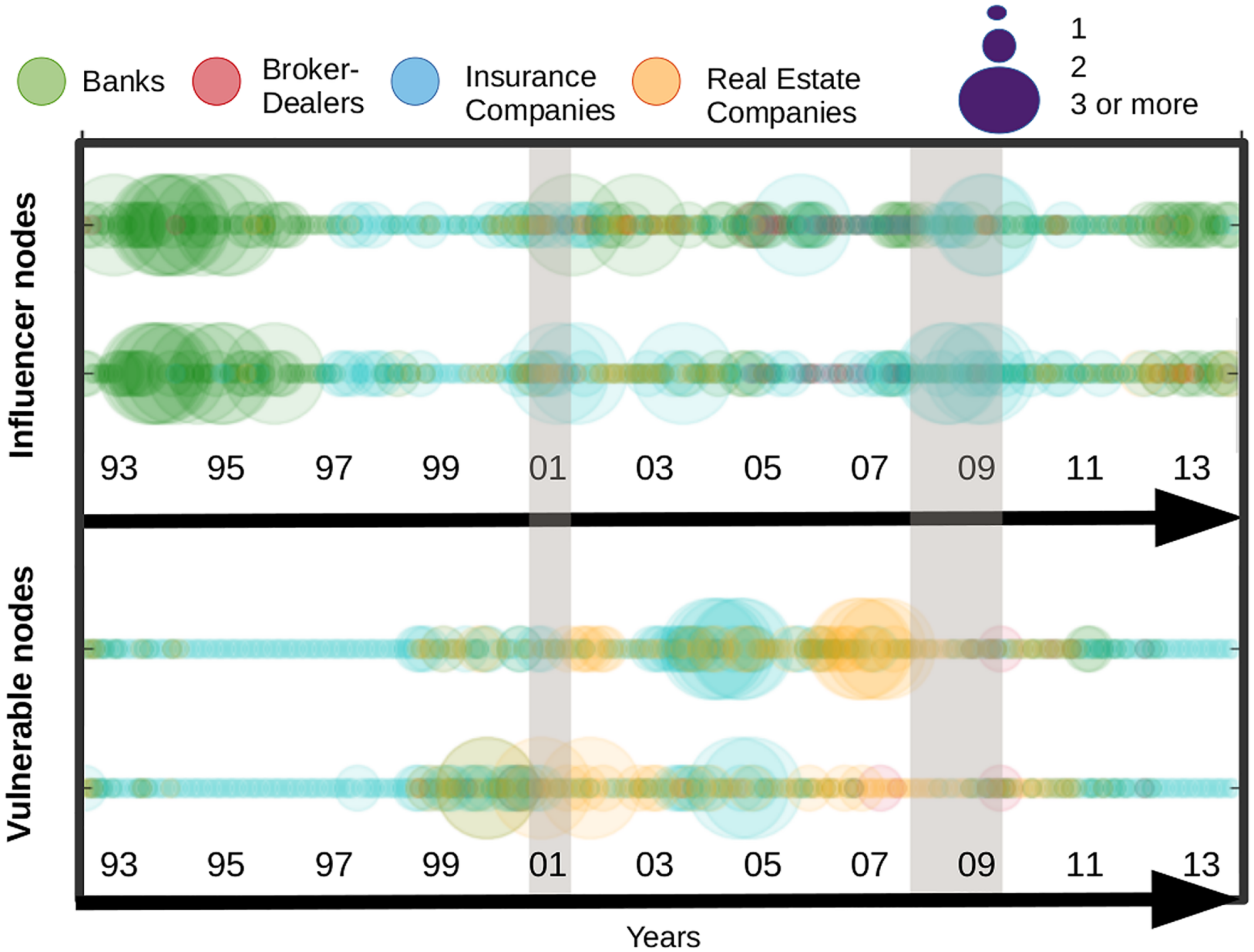
Most influencer and vulnerable nodes by means of a contagion process in the upper part, followed by the Katz centrality, from April 1993 to November 2014. The size of the circles represents the number of institutions with out-degree and in-degree among the top 20% highest values of the sample.

In Fig 12, we compare the results obtained by means of our contagion process with the Katz centrality measure. We stop the contagion process at the second iteration. For both measures, we count the number of institutions per sector which fall into the top 20% highest values. As explained in a previous section, the top 20% embeds the values ranging from the highest centrality value in a given month to a threshold which is equal to 80% of the highest value. This procedure is repeated at every period to flag the most influential/vulnerable institutions over time. The circles in the top panel, display the influence of each sector for the contagion process. Those for the Katz centrality stand just below. A visual inspection of the two subfigures support the closeness of their information content as the patterns are very similar over the whole sample. We can nevertheless notice a few differences. Among them, we can cite the role of the banking sector before the 2001 crisis which is characterised as a substantial propagator of spillovers only when using the Katz centrality measure. Turning to nodes’ influence, we can see that both results again provide similar information: (i) the banking sector is highly influential from 1993 to 2001, (ii) the insurance companies along with banks have emerged over time as key transmitters in the financial industry.

### Temporal measurements

**Fig 13:**
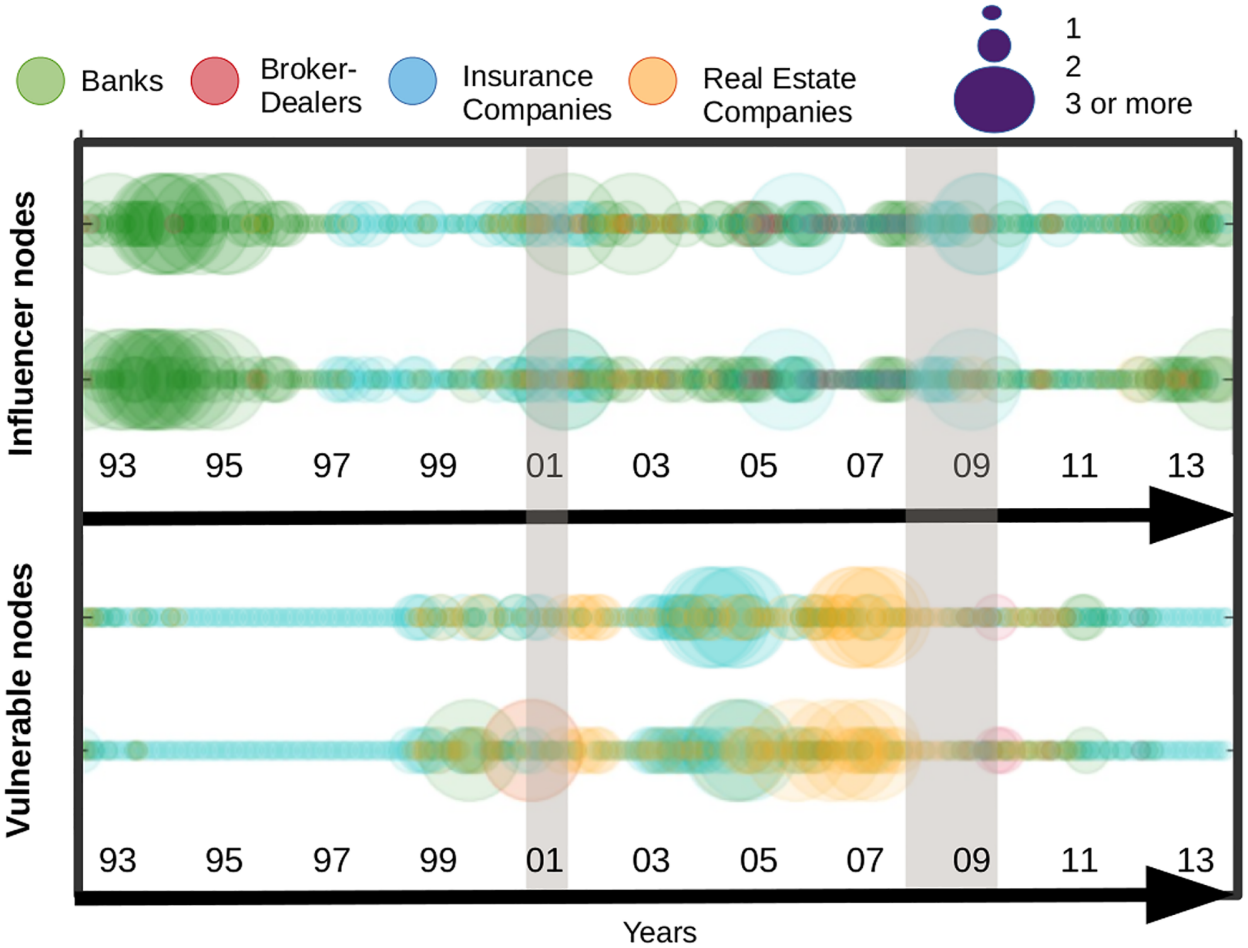
Most influencer and vulnerable nodes by means of an instantaneous contagion process (static 2-reach centrality measure), in the upper part, and a contagion process with time delay (temporal 2-reach centrality measure), in the lower part from April 1993 to November 2014. The size of the circles represents the number of institutions with out-degree and in-degree among the top 20% highest values of the sample.

More recently, a new line of research in network science has aimed to develop specific metrics for dynamic systems as opposed to standard metrics applied on successive static snapshots of time evolving networks. In accordance, we propose to apply as a last exercise a metric embedding information on the temporal sequence of edges and nodes. Fig 13 displays both temporal and static metrics. The former is computed by sector as the sum of top 20% most central institutions. We do it for both in-degree (i.e. “vulnerable” nodes) and out-degree (i.e. “influencer” nodes). We detail below how out-degrees and in-degrees are computed to account for time variation in the connections. Starting with in-degree, at each point in time, we first count for each node the number of incoming links at time *t*. We then count the number of incoming links at the previous period, i.e. at *t* − 1, of the connected nodes. We can view this measure as a modified version of the M-reach centrality in which first order connections and second order connections stem from networks at two successive time periods. By doing so, the metric features potential time delay in the contagion process and account for the sequence of appearance and disappearance of links over time. The reverse logic is applied for out-degrees. At time *t*, we count the number of outgoing links (i.e. connected nodes). By doing so, we identify for each node a set of influenced nodes (i.e. nodes from the network connected through outgoing links). Then, at *t* + 1, we count the number of links of the connected nodes. The spirit of such a measure is similar to the one of an infection process with time delay in which for instance first order neighbours are infected at time *t* considering the state of the network at time *t* and then second order neighbours are infected at time *t* + 1 considering the state of the network at time *t* + 1. Fig 13 also displays, above each temporal metric, the previous static version of the M-reach centrality metrics that is when we count the number of second order neighbors at fixed time period. We observe that both temporal and static metrics provide consistant information regarding the vulnerability and influence of sectors. We also note a few differences. A notable divergence for instance lies in the high concentration of vulnerable sectors in the run up to the 2007–2009 financial crisis that appears with the temporal metric and is less visible with the static one.

## Conclusion and discussion

Using a large set of tools from networks science, causality-based networks have been analysed in a large set of 155 financial institutions: all the banks, broker-dealers, insurance and real estate companies listed in the Standard & Poors’ 500 index during the period 1993–2014. In contrast to main body of research on financial networks, we pay particular attention to its temporal dimension by following the approached used in [6] designed to deal with its dynamic nature of financial institutions’ connections. Equipped with these dynamic causality-based networks, we describe its evolution per sector in the vein of [8] by using traditional tools from networks science such as centrality measures based on in-degree and out-degree as well as more advanced tools. The latter are intended to expend further traditional analysis by extracting information that was not attainable using simple sectoral centrality measures. Among the set of tools we are using, we can specifically emphasise measures of community and component structures as well as interface identification to offer a different view on the fragmentation/integration processes that took place over time in the US financial industry. We also apply an algorithm derived from the percolation theory to shed light on the question of influencing/vulnerable nodes or groups of nodes. Eventually, we propose a top institution representation drawing on the most highly connected institutions. By doing so, we can provide original empirical insight and tackle the following two objectives of the paper: (i) to provide new insights to network science by means of financial data, and (ii) to improve our understanding of the US financial industry over a long time span. Regarding our first objective, our work is one more attempt to construct a bridge between the physics thinking, in the spirit of simplicity, and equivalence between measurements. From the comparison of the different measures, our results tend to show that a large set of information can be extracted from the traditional in and out-degree centrality measures at three different levels: as (i) node-level, (ii) sectoral level, and (iii) by considering top values. Further information can be extracted from financial data by means of the communities’ and components’ structures. Eventually, our results suggest the quasi-equivalence on financial data of alternative measures as the one built on contagion process and the Katz centrality.

Turning to the second objective related to the systemic risk analysis, our results allow us to document four important patterns. First, banks have been highly influential since the early 1990s as documented by the temporal evolution of our normalized out-degree measurement as well as the contagion process. Second, real estate companies have been the most vulnerable sector in the financial industry especially over the 2001–2007 period. This is illustrated by in-degree centrality measurement and the (inverted) contagious process. Third, market integration drastically increased in the run-up to the 2007–2009 crisis either within the financial sector or between them. Component as well as community structures provide clear evidence on this feature. Fourth, the US financial industry has experienced a growing fragmentation from the crisis to the late 2013. This pattern appears clearly from the analysis of component and community structures. Finally, we applied a temporal measure, where the previously developed contagious process is applied but now over consecutive time windows. This last metric can be considered to study cascading failure mechanism.

Such results open up two important observations. First, it confirms in a dynamic context, that various tools from networks science can improve our knowledge of the financial system, stressing the need for further research in this direction. Second, the identification of a high and persistent level of integration across sectors calls into question the current sector-based approach to macroprudential surveillance.

This research can be extended in different ways. First, we rely in the whole study on an unweighted network as done in [8]. A natural extension would be to explore whether results are modified when additional information is embedded regarding the intensity of the links. Second, in line with a recent strand of the literature in network science and as illustrated in the last subsection of the analysis, using specific metrics dedicated to temporal network appears as a promising line of research to analyse financial systems.

## Supporting information

S1 Appendix Priors and posterior distribution simulation. (PDF)
