# Supplementary material for: Fragmentation, integration and macroprudential surveillance of the US financial industry: Insights from network science
Source: PLoS One. 2018 Apr 25;13(4):e0195110. doi: 10.1371/journal.pone.0195110 (PMC5919003; doi:10.1371/journal.pone.0195110)
Supplement: S1 Appendix — (PDF) [file pone.0195110.s001.pdf]

S1 Appendix

S1.A Priors

The various blocks of parameters are assumed to be independent,

$$p(\theta_0, \Sigma, Q) = p(\theta_0)p(\Sigma)p(Q).$$

The prior for the initial states of the time-varying coefficients, $f(\theta_0)$, is a Gaussian density,

$$\theta_0 \sim \mathcal{N}(\bar{\theta}, \overline{P}),$$

where $\bar{\theta}$ corresponds to the OLS point estimates of a training sample and $\overline{P}$ to four times the covariance matrix $\hat{V}(\bar{\theta})$. For both the simulation exercises and the empirical application, we use a training sample of 38 observations. For testing, $\bar{\theta}$ was estimated by restricting the parameters referring to the connections being tested to zero. On the other hand, the unrestricted estimates were used for $\hat{V}(\bar{\theta})$.

The prior for Q is inverse-Wishart,

$$Q \sim \mathcal{IW}(\overline{Q}^{-1}, T_0).$$

where T_0 is the prior degrees of freedom. Following [1], we set $T_0 = \dim(\theta_t)+1$ so that the prior is only weakly informative and the posterior for Q puts most weight on the data. Through a series of simulation exercises, [2] show that this configuration performs well in detecting different levels of time variation.

Following [3], we set $\overline{Q} = (0.01)^2 \times \hat{V}(\bar{\theta})$ multiplied by the number of observations in the training sample. Although moderately conservative, this choice is not expected to influence the results since the prior is dominated by the sample information.

Priors for Σ are set as

$$\Sigma \sim \mathcal{IW}(I_N, N+1),$$

where I_N is the identity matrix of size N. In the case of pairwise recursive testing with a bivariate TVP-VAR, a two by two identity matrix would be used and the degrees of freedom would be set to two instead of N.

S1.B Posterior distribution simulation

For any vector x_t, denote x^T the vector collecting the history of x_t up to period T. Thus, $x^T = [x_1', \ldots, x_T']'$.

We simulate the joint posterior distribution by sequentially drawing from the condtional posterior of the three blocks of parameters: the coefficients θ^T and the variance-covariance matrices Q and Σ.

S1.B.1 Parameter states, $\theta^{\mathbf{T}}$

The conditional distribution of the TVP-VAR parameters, θ^T, can be expressed as:

$$p(\theta^T \mid R^T, Q, \Sigma) = p(\theta_T \mid R^T, Q, \Sigma) \prod_{t=1}^{T-1} p(\theta_t \mid \theta_{t+1}, R^T, Q, \Sigma) \qquad (1)$$

Given the prior assumptions above and the state-space model, the conditional densities are normal and can be simulated using the algorithm proposed by [4].

Precisely, we can compute their means and variances through the forward and backward recursions of the Kalman filter and smoother. The last iteration of the filter provides the mean and variance for the first term in (1),

$$p(\theta_T \mid R^T, Q, \Sigma) = \mathcal{N}(\theta_{T|T}, P_{T|T})$$

A draw from the distribution is used in the backward recursions to simulate the remaining terms in (1). Conditional on the information in θ_{t+1}, θ_t is conditionally normal with mean and variance given respectively by,

$$\begin{aligned} \theta_{t|t+1} &= \theta_{t|t} + P_{t|t} P_{t+1|t}^{-1} (\theta_{t+1} - \theta_{t|t}), \\ P_{t|t+1} &= P_{t|t} - P_{t|t} P_{t+1|t}^{-1} P_{t|t} \end{aligned}$$

The backward recursions draw sequentially $\theta_{T-1}, \theta_{T-2}, \ldots, \theta_1$ from the conditional distribution

$$p(\theta_t \mid R^T, Q, \Sigma) = \mathcal{N}(\theta_{t|t+1}, P_{t|t+1}), \qquad (2)$$

in order to generate a random trajectory θ^T.

S1.B.2 Innovation variance-covariance matrix, Q

Conditional on a realization of θ^T, the TVP-VAR parameter innovations, v_t, are observable. Under the linear transition law, v_t is i.i.d. normal. Given the natural conjugate prior specified above, the posterior is inverse-Wishart,

$$p(Q \mid R^T, \theta^T) = \mathcal{IW}(Q_1^{-1}, T_1),$$

with scale and degree-of-freedom parameters,

$$Q_1 = \overline{Q} + \sum_{t=1}^{T} v_t v_t' \qquad T_1 = T_0 + T.$$

S1.B.3 Residual variance-covariance matrix, Σ

Conditional on a realization of θ^T, the TVP-VAR residuals, u_t, are observable. Given the conjugate prior assumption given above, the conditional posterior density of Σ is given by

$$p(\Sigma \mid R^T, \theta^T) = \mathcal{IW}(\Sigma_1^{-1}, N_1),$$

where,

$$\Sigma_1 = I_N + \sum_{t=1}^{T} u_t u_t' \qquad N_1 = N + 1 + T.$$

For each estimation, we perform 6000 iterations of the Gibbs sampler and discard the first 1000 draws. We then keep only the 5th of every draw in order to mitigate autocorrelation among draws. The remaining sequence of 1000 draws forms a sample of the joint posterior distribution $p(\theta^T, Q, \Sigma \mid R^T)$. We use this to estimate Bayes factor and test the time-varying hypothesis of no connection between two nodes of the system.

References

[1] Cogley T. How fast can the new economy grow? A Bayesian analysis of the evolution of trend growth. Journal of Macroeconomics. 2005;27:179–207.

[2] Reusens P, Croux C. Detecting time variation in the price puzzle: An improved prior choice for time varying parameter VAR models; 2014.

[3] Primiceri G. Time Varying Structural Vector Autoregressions and Monetary Policy. Review of Economic Studies. 2005;72(3):821–852.

[4] Carter CK, Kohn R. On Gibbs Sampling for State Space Models. Biometrika. 1994;81(3):541–553.
